# A "Milk Epidemic" of Scarlet Fever

**Published:** 1901-11-23

**Authors:** 


### A "MILK EPIDEMIC" OF SCARLET FEVER.

A REPORT recently issud by the Medical Officer of Health for the county of London, giving an account of an extensive outbreak of scarlet fever in Shoreditch and Bethnal Green which was traced to the milk supply, shows two things: first the danger of using milk from an infected source, and second the inefficiency of the powers entrusted to sanitary authorities for the prevention of such danger. We cannot here attempt to give even a condensed account of the manner in which the infection was tracked through the vendors to a particular middleman and through him to churns coming from certain parts of Staffordshire and Derbyshire, and then by aid of the medical officers of these counties and those of the half-dozen districts involved to one particular farm on which persons were found to be suffering from sore throat and rash, which in one case at least was followed by definite desquamation. Suffice it to say that the whole story redounds greatly to the credit of Mr. Shirley Murphy, Dr. Hamer and the several district medical officers. The point of importance, however, is to be found in the almost insuperable difficulties which were thrown in their way by the fact that there was no immediate and direct means of tracing the origin or the distribution of the milk by which the infection had been carried. When milk has once been mixed it is of course impossible to trace it, but in this case more than half of the milk distributed by Mr. X. (the dealer implicated) was delivered to the local vendors in the form of "whole churns," and it would have been a simple matter for a record of their origin and destination to have been kept. The diagrams which accompany the report show the number of attacks occurring and the number of houses invaded day by day among those supplied by Mr. X. and among persons supplied by vendors who did not obtain their milk from this contractor, and they demonstrate in a most striking manner, how immediate an effect was produced among the former, as compared with the latter, by stopping the implicated milk supply. Mr. Murphy, in summing up the results of his investigation says that the history of this outbreak shows the need for amendment of the law with regard to outbreaks of infectious disease attributed to milk. In this case the contractor, acting on Mr. Murphy's advice, stopped the distribution of the milk some days before it would have been possible to compel this course if legal power had had to be sought. The great difficulty, however, arose not so much in tracing the milk to the contractor, but in tracking it, through him, and through the multitude of churns which he received, to the particular farm where the disease existed. It was known that the milk distributed by Mr. X. was infected, but it was not known in which churns the infection lay. As a matter of fact local vendors who had been warned and urged by the medical officers of health to change their supplies were content to leave it to Mr. X. to send them milk of different origin from that which they had hitherto been receiving, and he, not knowing which was the infected milk, met their wishes by making alterations in the allotment of the churns to particular vendors. The infected churns merely went to somebody else, sometimes into other districts, as was shown to have happened in several instances. The serious point is that under the present state of the law "the medical officers of health of other London districts if they had known of the danger with which their districts were threatened could not have prevented it, and the County Council had no jurisdiction in regard to such a matter." Clearly some modification of the law is wanted in the direction of compelling the dealers to keep such a record of their churns as will render it possible to trace their source and distribution without delay.

